# Risk Factors and Outcome of *C. difficile* Infection after Hematopoietic Stem Cell Transplantation

**DOI:** 10.3390/jcm9113673

**Published:** 2020-11-16

**Authors:** Chiara Rosignoli, Giuseppe Petruzzellis, Vera Radici, Gabriele Facchin, Marco Girgenti, Rossella Stella, Miriam Isola, Martalisa Battista, Alessandra Sperotto, Antonella Geromin, Michela Cerno, Alessandra Arzese, Paola Deias, Carlo Tascini, Renato Fanin, Francesca Patriarca

**Affiliations:** 1 Clinica Ematologica ed Unità di Terapie Cellulari, Azienda Sanitaria Universitaria Friuli Centrale, Piazzale S. Maria della Misericordia 10, 33100 Udine, Italy; chiara.rosignoli@edu.unife.it (C.R.); giuseppe.petruzzellis93@gmail.com (G.P.); vera.radici@hotmail.it (V.R.); gabriele.facchin.92@gmail.com (G.F.); marco.girgenti@icloud.com (M.G.); stellarossella@gmail.com (R.S.); martalisa.battista@asufc.sanita.fvg.it (M.B.); alessandra.sperotto@asufc.sanita.fvg.it (A.S.); antonella.geromin@asufc.sanita.fvg.it (A.G.); michela.cerno@asufc.sanita.fvg.it (M.C.); paola.deias@asufc.sanita.fvg.it (P.D.); renato.fanin@asufc.sanita.fvg.it (R.F.); 2 Istituto di Statistica, Dipartimento di Area Medica, Università di Udine, 33100 Udine, Italy; miriam.isola@uniud.it; 3 SOC Microbiologia, Azienda Sanitaria Friuli Centrale, 33100 Udine, Italy; alessandra.arzese@uniud.it; 4 Dipartimento di Area Medica, Università di Udine, 33100 Udine, Italy; 5 SOC Malattie Infettive, Azienda Sanitaria Universitaria Friuli Centrale, 33100 Udine, Italy; carlo.tascini@asufc.sanita.fvg.it

**Keywords:** *C. difficile* infection, autologous stem cell transplantation, allogeneic stem cell transplantation, risk factors, outcome

## Abstract

Patients who undergo hematopoietic stem cell transplants (HSCT) are at major risk of *C. difficile* (CD) infection (CDI), the most common cause of nosocomial diarrhea. We conducted a retrospective study, which enrolled 481 patients who underwent autologous (220) or allogeneic HSCT (261) in a 5-year period, with the aim of identifying the incidence, risk factors and outcome of CDI between the start of conditioning and 100 days after HSCT. The overall cumulative incidence of CDI based upon clinical evidence was 5.4% (95% CI, 3.7% to 7.8%), without any significant difference between the two types of procedures. The median time between HSCT and CDI diagnosis was 12 days. Out of 26 patients, 19 (73%) with clinical and symptomatic evidence of CDI were positive also for enzymatic or molecular detection of toxigenic CD; in particular, in 5 out of 26 patients (19%) CD binary toxin was also detected. CDI diagnoses significantly increased in the period 2018–2019, since the introduction in the microbiology lab unit of the two-step diagnostic test based on GDH immunoenzymatic detection and toxin B/binary toxin/027 ribotype detection by real-time PCR. Via multivariate analysis, abdominal surgery within 10 years before HSCT (*p* = 0.002), antibiotic therapy within two months before HSCT (*p* = 0.000), HCV infection (*p* = 0.023) and occurrence of bacterial or fungal infections up to 100 days after HSCT (*p* = 0.003) were significantly associated with a higher risk of CDI development. The 26 patients were treated with first-line vancomycin (24) or fidaxomicine (2) and only 2 patients needed a second-line treatment, due to the persistence of stool positivity. No significant relationship was identified between CDI and the development of acute graft versus host disease (GVHD) after allogeneic HSCT. At a median follow-up of 25 months (range 1–65), the cumulative incidence of transplant related mortality (TRM) was 16.6% (95% CI 11.7% to 22.4%) and the 3-year overall survival (OS) was 67.0% (95% CI 61.9% to 71.6%). The development of CDI had no significant impact on TRM and OS, which were significantly impaired in the multivariate analysis by gastrointestinal and urogenital comorbidities, severe GVHD, previous infections or hospitalization within two months before HSCT, active disease at transplant and occurrence of infections after HSCT. We conclude that 20% of all episodes of diarrhea occurring up to 100 days after HSCT were related to toxigenic CD infection. Patients with a history of previous abdominal surgery or HCV infection, or those who had received broad spectrum parenteral antibacterial therapy were at major risk for CDI development. CDIs were successfully treated with vancomycin or fidaxomicin after auto-HSCT as well as after allo-HSCT.

## 1. Introduction

*Clostridium difficile* is a Gram-positive, anaerobic, spore-forming, toxin-producing bacillus, that represents the most common cause of nosocomial diarrhea [1,2]. Clinical manifestations range from moderate diarrhea to life-threatening pseudomembranous colitis.

The bacterium is present in the environment and its spores are transmitted by the fecal–oral route. Common risk factors for CD infection (CDI) are antibiotic exposure, older age and hospitalization. Patients who undergo hematopoietic stem cell transplants (HSCT) are at a more major risk of CDI than other subjects, due to prolonged hospitalization, multiple infections with massive usage of antibiotic therapy [3], alteration of the intestinal mucosa caused by chemotherapy during conditioning regimens, and immunosuppressive treatment [4,5]. The role of CDI during HSCT remains unclear due to studies with heterogeneous patient cohorts and conflicting results [6,7,8,9,10,11,12,13,14,15,16] (Table 1). The risk for CDI among these patients is reported to be frequently higher than in the general population [9,16]. However, serious and life-threatening disease courses seem to be rare [8,11]. Another possibly severe complication in patients undergoing allogeneic HSCT is acute graft versus host disease (GVHD), which appears closely related to the infection through a bidirectional cause–effect link [6,17].

In order to identify the incidence, risk factors and outcome of CDI after HSCT we conducted a retrospective study, which enrolled 481 patients who underwent autologous or allogeneic HSCT in a 5-year period in a single Transplant Center.

## 2. Methods

### 2.1. Patients

This retrospective study included 481 patients who underwent HSCT at the Transplant Center of Udine between 1 January 2015 and 31 December 2019; 261 received allogeneic HSCT (allo-HSCT) and 220 underwent autologous HSCT (auto-HSCT). The patient and transplant features are summarized in Table 2. Upon the admission to the transplant center, all patients signed a consent form where they expressed their consent to the collection of their medical data for scientific studies. This consent form was approved by our Ethics Committee. Median age at the time of the procedure was 55 years (range 15–75). The most common underlying hematological diseases were acute leukemias (41%), followed by multiple myeloma (30%) and lymphomas (24%). The stem cell source was peripheral blood for 96% of patients. A reduced-intensity conditioning regimen (RIC) was chosen in 13% of patients [6]. A high-resolution HLA-typing for all major histocompatibility complex (MHC) Class I loci (HLA-A, HLA-B, HLA-C) and for the MHC Class II HLA-DRB1 locus was required. Stem cells came from an HLA-matched sibling donor, haploidentical donors, matched unrelated and mismatched unrelated donors in 19%, 18%, 40% and 23% of allo-HSCTs, respectively. For allo-HSCT patients, GVHD prophylaxis consisted of cyclosporine or tacrolimus plus 3 to 4 methotrexate courses for HLA-matched sibling transplants, with the addition of antithymocyte globulin for unrelated transplants; tacrolimus, mycophenolate mofetil and post-transplant cyclophosphamide was the GVHD prophylaxis for HSCT from haploidentical donors. The median length of hospitalization was 45 days (range 21–146) after allo-HSCT, and 34 days (range 18–51) after auto-HSCT. Among the patients undergoing allo-HSCT, acute GVHD was graded according to the 1991 Consensus Conference on Acute GVHD Grading criteria [18].

We examined the two months prior to the admission to the Transplant Unit and we recorded the occurrences of infections, hospitalizations and the need of broad-spectrum parenteral antibiotic therapy. Thirty-seven per cent of patients had developed one or more infections within two months before HSCT diagnosed by bacterial and mycotic isolates in blood, broncholavage liquid or other fluids and/or by radiological findings. Mycotic infections were included if they could be categorized as probable or proven mycoses according EORTC classification [19]. Fourteen per cent of patients had a history of previous infections caused by multidrug-resistant (MDR) bacteria, which included methicillin-resistant *Staphylococcus aureus*, carbapenemase-resistant *Klebsiella pneumoniae*, carbapenemase-resistant *Enterobacteriacae*, vancomycin-resistant *Enterococcus faecium*/*faecalis* and carbapenemase-resistant *P. aeruginosa*. Thirty-two per cent of patients needed hospitalization within two months before HSCT, because of treatment for the underlying hematological disease or clinical complications. Forty per cent of patients were treated with broad spectrum parenteral antibacterial therapy according to the clinical guidelines exposed in the following paragraph.

At transplant, two hundred and four patients (42%) had gastrointestinal and/or urogenital comorbidities, which included gallbladder stones, diverticulosis, haemorrhoids, colon adenomas or cancer, intraductal papillary mucinous neoplasms (IPMN) of pancreas, bladder prolapse, chronic gastritis, perianal abscesses or fistulae, gastrointestinal reflux, prostatic hypertrophy and prostatitis. The hematopoietic cell transplantation–comorbidity Iindex (HCT-CI) was equal or greater than 3 in 228 patients (47%). Previous abdominal surgery within 10 years before HSCT was reported in 172 patients (36%) and was represented by appendicectomy, bowel resections, gallbladder resections, surgery of any type of intestinal herniation, nephrectomy, annexectomy and hysterectomy, prostatic resections, laparoscopic or laparotomic explorations.

### 2.2. Monitoring and Management of Infections

Before admission to the Transplant Unit, and therefore starting conditioning, all patients performed throat, nasal and rectal swabs, and *C. difficile* detection in stool as a screening test, in order to detect colonization by MDR bacteria and by CD, respectively. The microbiological method for CD detection was changed during the 5-year study; between 2015 and 2017, GDH and direct detection of CD toxin A and B in stools by enzyme linked immune assay (Premier toxins A&B, Meridian Bioscience, Cincinnati, OH, USA) was applied as diagnostic tests, but from January 2018, a two-step algorithm was applied, based on GDH detection by enzyme linked chemiluminescent assay (CLIA) (LIAISON *C. difficile* GDH, Diasorin, I) as a reflex test, and a real-time polymerase chain reaction (PCR) for the detection of toxin B, binary toxin and 027 ribotype test (Xpert *C. difficile*, Cepheid, Sunnyvale, USA) as a confirmatory test for the identification of toxigenic CD.

All patients had individual rooms with high efficiency particulate air (HEPA) filtration and positive pressure flow. The conditioning regimen was administered through a central venous catheter. The patients received proton pump inhibitors. During the aplasia phase anti-infective prophylaxis consisted of Levofloxacine and Valaciclovir/Acyclovir for all patients, with the addiction of an azole or liposomial Amfotericine B after allo-HSCTs. After engraftment, the prevention of *Pneumocystis jirovecii* infection was based on trimetoprim-sulfamethoxazole or pentamidine aerosol in case of allergy to the sulphonamides. Neutropenic fever was treated with piperacillin-tazobactam plus vancomicin followed by meropenem as second-line antibiotic therapy, while waiting for results of blood and urine cultures. Drug dosage was driven by therapeutic drug monitoring in the blood and according to renal and hepatic functions and drug interactions.

### 2.3. Monitoring and Management of CDI

All patients with three or more episodes of unformed stools with or without abdominal pain, nausea and vomiting or fever between start of conditioning and +100 days after transplant were tested for the presence of *C. difficile* in stools and underwent three sets of blood cultures by peripheral veins and central catheters in case of fever. Stool samples were also evaluated for other bacterial, fungal and viral pathogens. A case of CDI was defined as a patient showing the above mentioned signs and symptoms of the infection supported by a *C. difficile* positivity test in stool [20,21]. The frequency and severity of diarrhea were retrospectively evaluated with physician and nurse notes. The severity of the gastrointestinal symptoms was graded according to National Cancer Institute Common Terminology Criteria for Adverse Event (CTCAE 3.0) [22].

### 2.4. CDI Treatment

CDI was treated in accordance with international guidelines [20,21]. In addition to the patient being isolation in a single room with HEPA filtration, all the hospital staff and visitors had to use additional contact precautions (gloves and coat). Oral vancomycin was used as first-line treatment. A course of fidaxomicin was reserved to patients who had received highly immunosuppressant GVHD prophylaxis for mismatched unrelated or haploidentical donors. Negativization of CD was evaluated on a stool sample collected after clinical improvement and at least seven days of therapy. In the case of persistence of stool positivity, a course of fidaxomicin was administered.

### 2.5. Statistical Analyses

Data were collected in an XLS Database (Microsoft office 2016; Microsoft Corporation, Redmond, WA, USA) and subsequently imported into STATA16 for Windows 10. The close-out for data collection was 31 December 2019. Transplant-related mortality (TRM) was defined as death due to all causes not related to the underlying hematological disease, while overall survival (OS) was defined as the time in months from transplantation to either death or last follow-up of the patients. OS was described using the Kaplan–Meier method, and a log-rank test was used to compare different groups. The cumulative incidence method was used to estimate TRM and CDI incidence accounting for the presence of competing risks. The competing event for TRM was relapse of disease, while the competing event for CDI was death from any cause. We analyzed patient-related and transplant-related variables and their possible relationships with incidence of CDI, TRM and OS. The patient variables are: age (as continuous variable), underlying hematological disease, gastrointestinal and urogenital comorbidities, history of infections, history of infections from MDR bacteria, HCT-CI, history of previous abdominal surgery, HBV and HCV infections, hospitalization up to two months before transplant, IgG value less than 700 mg/dL, administration of antibiotic therapy up to two months before transplants, CD infection or colonization before HSCT, and disease status at HSCT. The transplant-related variables are type of HSCT, type of conditioning (total body irradiation-containing regimens or Busulfan-containing regimens or RIC regimens or other miscellaneous conditioning mainly applied for auto-HSCT), number of antibiotic lines used during hospitalization, mucositis development and grading, use of total parenteral nutrition (TPN), incidence of acute GVHD and grading, CMV-DNAemia occurrence, and other main infections after HSCT. A univariate and a multivariate backward stepwise competing risk regression were used to identify which variables were associated with incidence of CDI, TRM and OS. This model is based on the hazard of the sub-distribution and provides a simple relationship between covariates and cumulative incidence. Multivariate analyses considered all variables significant at *p* ≤ 0.10 in the univariate analysis.

## 3. Results

### 3.1. Clinical Characteristics of CD Infections

**Figure 1:**
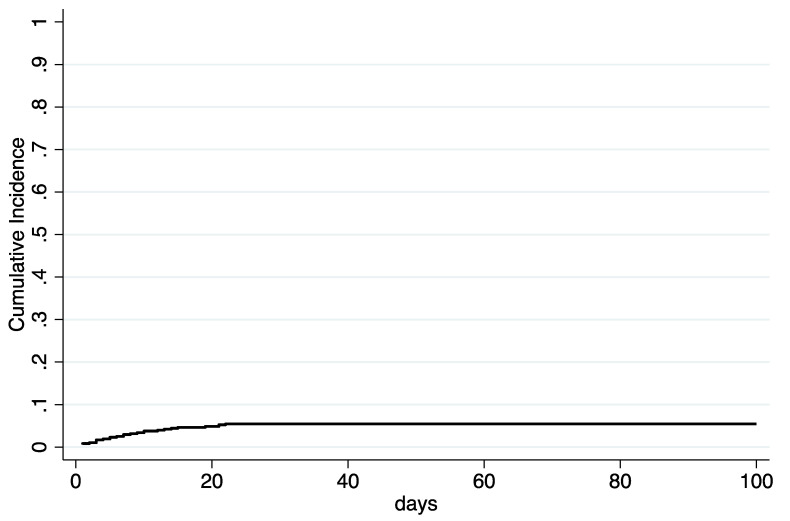
Cumulative incidence of CDI after HSCT. CDI = clostridium difficile infection; HSCT = hematopoietic stem cell transplantation.

**Figure 2:**
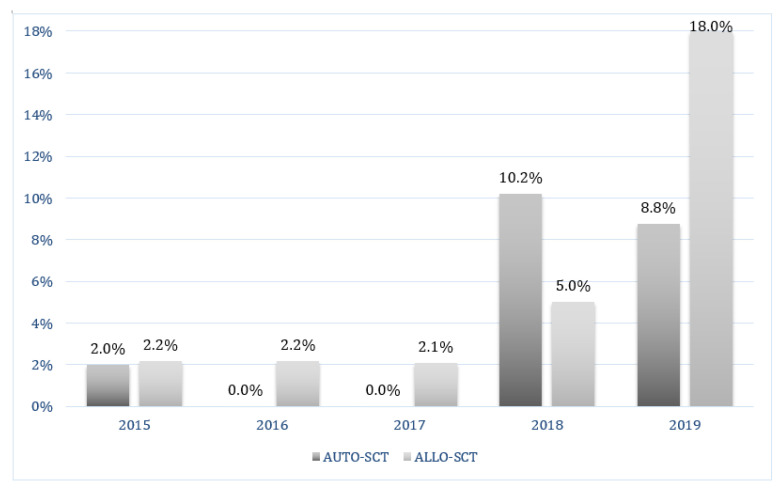
CDI cases for years. CDI = clostridium difficile infection; SCT = stem cell transplantation.

Upon admission at the Transplant Unit, screening for CD and MDR bacteria was negative in all patients. Twenty-five patients had a history of previous CDI within 12 months before HSCT. Of 481 patients who underwent HSCT, 127 (26%) presented diarrhea during the hospitalization, and in 26 of the symptomatic patients (20%) CDI was diagnosed (Table 3). None of them were recurrences of a previous CDI. The overall cumulative incidence of CDI was 5.4% (95% CI, 3.7% to 7.8%) (Figure 1). CDI diagnoses increased over time; in fact, 4 out of 265 patients (1.5%) developed CDI between 2015 and 2017, in comparison with 22 out of 216 patients (10.2%) in the period 2018–2019 (*p* = 0.000) (Figure 2).

In total, 11 patients had received auto-HSCT and 15 had undergone allo-HSCT. The cumulative incidence of CDI was 5.4% (95% CI 3.67% to 7.77%) after allo-HSCT, and 4.2 (95% CI 2.6% to 6.3%) after auto-HSCT, with no significant difference between the two types of procedures. Out of 26 patients, 19 (73%) were also positive for toxigenic *C. difficile*. In particular, 5 out of 26 patients (19%) appeared to be infected by toxin B and binary toxin positive CD strains, as detected by real-time PCR. All patients presented diarrhea with abdominal pain, and eight of them (30%) showed concomitant fever; no cases of ileus were observed. The median time between HSCT and CDI diagnosis was 12 days after HSCT (range −5 to +100). Fourteen CDIs (57%) occurred during severe neutropenia (neutrophil count < 1 × 10^9^/L). Gallbladder stones were the most common gastrointestinal or urogenital comorbidity among patients with CDI (23%), followed by benign prostatic hypertrophy (19%), chronic gastritis and gastro-esophageal reflux (15%), history of perianal infections (11%), benign and malignant cancer of female reproductive system (11%), adenomas or cancer of colon (7%), inguinal hernia (7%), bladder prolapse (3%) and kidney cancer (3%). The most frequent previous abdominal surgery was cholecystectomy (15%), followed by appendectomy (11%), annexectomy and hysterectomy (11%), perianal surgery (11%), inguinal reduction (7%), bowel surgery (7%), nephrectomy (3%), emergency laparotomy (3%) and transurethral resection of the prostate (3%).

### 3.2. Risk Factors for CDI Development

Table 4 reports the univariate analysis of the factors associated with CDI incidence. Among patient-related factors, age > 60 years (*p* = 0.03), gastrointestinal and urogenital comorbidities (*p* = 0.01), infections diagnosed within two months before HSCT (*p* = 0.03), previous abdominal surgery (*p* = 0.007), HCV infection (*p* = 0.003), hospitalization before HSCT (*p* = 0.02) and antibiotic therapy before HSCT (*p* = 0.000) are closely associated with CDI development after HSCT. About transplant-related factors, TPN use (*p* = 0.09), CMV reactivation (*p* = 0.05) and incidence of other infections after HSCT (*p* = 0.08) were significantly linked to CDI occurrence. However, transplant type (allogeneic or autologous), conditioning regimen or donor did not have any significant impact on CDI incidence. Moreover, no significant relationship was identified between CDI and development of acute GVHD. In fact, neither acute gastro-intestinal GVHD, nor grade III–IV acute GVHD were identified among allo-HSCT cases infected by CD. In the multivariate analysis (Table 5), previous abdominal surgery (*p* = 0.002), antibiotic therapy before SCT (*p* = 0.000), HCV infection (*p* = 0.023) and occurrence of other bacterial or fungal infections after HSCT (*p* = 0.003) were significantly associated with a higher risk of CDI development.

### 3.3. Outcome

The first-line treatment for CDI was vancomycin for 24 patients and fidaxomicin for 2 patients. Only two patients (8%), who had previously received vancomycin, needed a second-line treatment with fidaxomicin. Median time of stool negativization was 17 days (5–56). No CD recurrence was observed.

**Figure 3:**
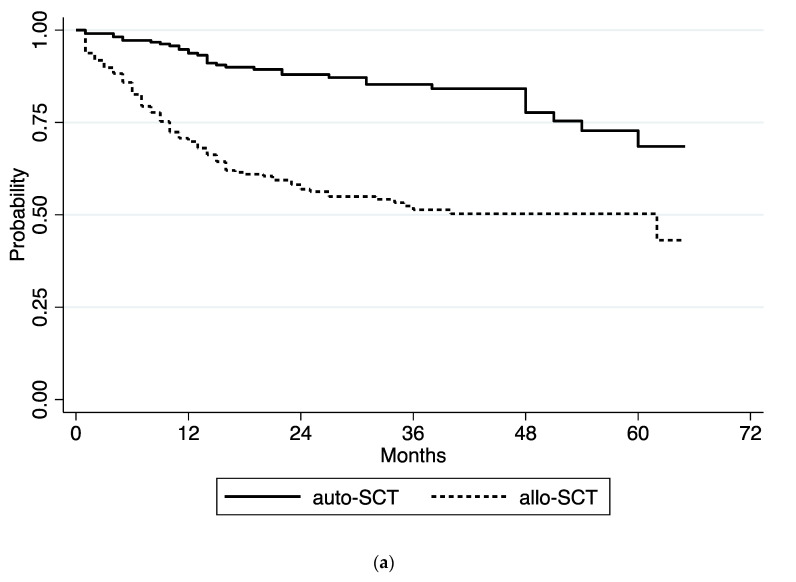

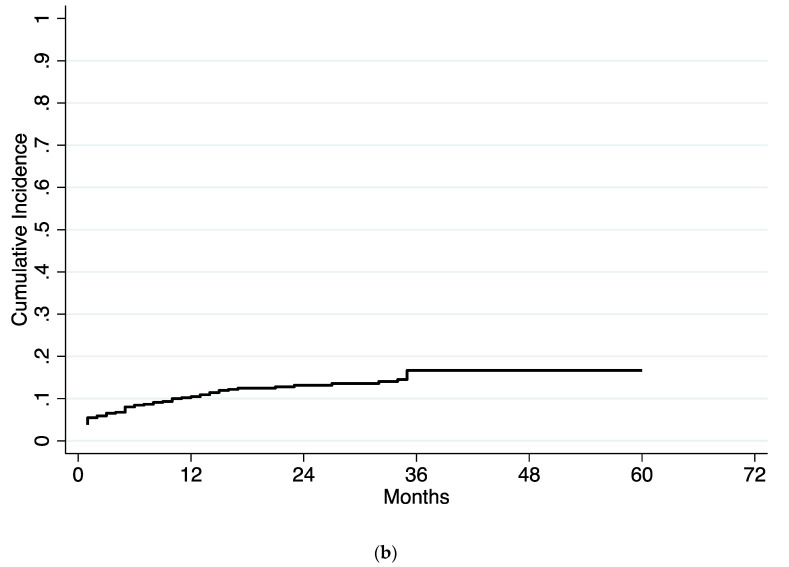
(**a**) Overall survival according to procedure; (**b**) Transplant-related mortality in all study population.

At a median follow-up of 25 months (range 1–65) after HSCT, 334 out of 481 patients (69%) were alive, while 147 patients (31%) died; 57 from transplant-related complications and 90 due to relapse of the underlying hematological disease. Cumulative incidence of TRM was 16.6% (95% CI 11.7% to 22.4%) (Figure 3b). The one-year TRM was 10.2% (95% CI 7.7% to 13.1%) after allo-HSCT and 6.5% (95% CI 4.5% to 8.9%) after auto-HSCT. The most common causes of death were infections (75%), followed by GVHD (16%), hemorrhages (4%), organ toxicities (3%) and unknown causes (2%). In the allo-HSCT group, 118 of 261 patients (45%) developed acute GVHD, which was grade I–II in 106 patients (40%) and grade III–IV in 12 patients (5%). Of these 12 patients, 7 died because of GVHD. In the univariate analysis the factors that significantly increased TRM were history of leukemia in comparison with myeloma or lymphoma (*p* = 0.007), allo-HSCT in comparison with auto-HSCT (*p* = 0.000), gastrointestinal and urogenital comorbidities (*p* = 0.004), history of infections before HSCT (*p* = 0.012), history of MDR infections before HSCT (*p* = 0.013), previous hospitalization (*p* = 0.007), active disease at transplant (*p* = 0.019), mismatched unrelated donor (*p* = 0.008), more than two antibiotic therapy lines after HSCT (*p* = 0.030), use of TPN (*p* = 0.030), grade IV acute GVHD (*p* = 0.000), and other infection after HSCT (*p* = 0.000). However, the development of CDI had no significant impact on TRM (*p* = 0.181). In the multivariate analysis, gastrointestinal and urogenital comorbidities (*p* = 0.008) and severe GVHD (*p* = 0.001) were significantly associated with TRM increase.

The 3- and 5-year OS were 67.0% (95% CI 61.9% to 71.6%) and 58.5% (95% CI 50.9% to 65.4%), respectively. As expected, patients who underwent auto-HSCT showed a significantly longer OS [3-year OS of 85.3% (95% CI 78.9% to 89.8%)] in comparison to those who received allo-HSCT (3-year OS 51.4% (95% CI 44.1% to 58.2%)) (*p* = 0.000) (Figure 3a).

In the univariate analysis, the significant factors for OS impairment were history of previous infections (*p* = 0.000), history of MDR infections (*p* = 0.001), abdominal surgery (*p* = 0.011), previous hospitalization (*p* = 0.000), antibiotic therapy before HSCT (*p* = 0.000), active disease at transplant (*p* = 0.000), HCT-CI ≥ 3 (*p* = 0.000), antibiotic therapy post-HSCT (*p* = 0.033), and other infections post-HSCT (*p* = 0.000). Female sex (*p* = 0.009), auto-HSCT procedure (*p* = 0.000) and diagnosis of myeloma (*p* = 0.000) were all variables significantly associated with longer OS. The development of CDI was not associated with a significant impairment of OS (*p* = 0.23). The multivariate analysis for OS showed that previous infections (*p* = 0.024), previous hospitalization (*p* = 0.003), active disease at transplant (*p* = 0.000) and infections post-HSCT (*p* = 0.043) are significant risk factors impairing OS. Multivariate analysis for TRM and OS is summarized in Table 6.

## 4. Discussion

CDI is a well-known major cause of diarrhea in transplanted patients [23]. In our study, 20% of all patients who complained of diarrhea during hospitalization showed *C. difficile* positivity in stool samples with an overall cumulative incidence of 5.4%, which is lower than the range between 9% and 24% previously reported (Table 1).

Moreover, we observed no significant difference in the CDI incidence between patients who had received auto-HSCT or allo-HSCT, confirming what was reported by Bruminhent et al. [10], while in other studies CDI was more frequent after allo-HSCT [6,9]. Most of our cases of CDI were diagnosed in the 2018–2019 period, since the diagnostic two-step algorithm, including screening by GDH followed by confirmatory testing for toxins with real-time PCR, was applied. Therefore, the lower CDI incidence observed in our case-series in comparison with previous studies could be related to the employment of a less sensitive method in the first three years of the study, together with a less stringent selection of samples for testing. Moreover, our study confirmed that a two-stage algorithm is a reliable method for CDI diagnosis in symptomatic patients [20,21]. As expected, in our study CDI occurred in the early post-transplant period at a median time of 12 days after HSCT, similarly to the median range between 8 and 38 days previously reported in the literature [6,9]. These data underline the fact that the bacterium is probably presents in the fecal microbiota of patients at admission to the Transplant Unit, and its growth can be promoted by bowel dysbiosis caused by previous prolonged hospitalizations, treatments with chemotherapy or antibiotics, bowel infections and unbalanced diet. Jain et al. [12] identified previous toxigenic CD colonization as a risk factor for CDI development after transplants; as in our experience we did not identify any CD carriers at the time of admission, this risk factor cannot be compared between our data and previous studies. In our study the main risk factors for CDI development were antibiotic therapy before HSCT, previous abdominal surgery, HCV infection, and other bacterial and fungal infections identified before or concomitantly with CDI during hospitalization for HSCT. Therefore, the use of antibiotics before and during HSCT hospitalization remains the leading risk factor in our analysis as well as in previous studies [6,7,15]. Broad spectrum antibiotics, such as cephalosporines, penicillines and fluoroquinolones, are associated with gut dysbiosis, development of bacterial resistance, malabsorption and alteration of the gut–liver axis [3]. In fact, antibiotic administration might cause the overall reduction of gut microbiota, particularly the obligate anaerobic species; it is interesting to report that *Clostridium scindens* could be capable of inhibiting CD growth [3,24] by decreasing the metabolism of primary bile acids to secondary bile acids. In our study, previous abdominal surgery was a significant risk factor for CD development. Even if this clinical association was observed by other authors [25], the pathogenesis is still unclear. In our study, the most common gastrointestinal surgery was cholecystectomy after history of gallbladder stones, suggesting that the increase in primary bile acids production and the alteration in metabolism might facilitate the germination of CD spores in the bowel [26]. Moreover, any type of abdominal surgery could modify the gut microbiota [27,28], changing anatomy and creating a new microbiological microenvironment. It has to be highlighted that in our study all the significant risk factors for CDI were patient-related and were strictly linked to medical history, comorbidities, complications of chemotherapy and infections, while all the transplant-related variables, such as conditioning and choice of donors for allo-HSCT recipients, had no significant impact on the risk of CDI development. Another controversial point is the link between GVHD and CDI. In our analysis, the frequency of CDI was similar in patients with or without acute GVHD, as reported by other authors [15,19]. However, other studies [6,9,16] observed that CD was a significant risk factor for acute GVHD development, in particular for the cases involving the gastro-intestinal system, suggesting that gut dysbiosis and disruption of mucosal barriers might promote CDI as well as GVHD. Moreover, GVHD itself was reported as a risk factor for CDI, since some patients with severe acute GVHD showed concomitant CDI, with could complicate the clinical course further.

In our study all CDIs successfully responded to first- or second-line treatments, and there were no recurrences. As most studies show, CDI has no significant impact on TRM and OS. However, it is interesting to note that events occurring within two months before HSCT, such as hospitalization, infections and need of broad spectrum parenteral antibacterial therapy, increased the risk for CDI development, as well as impaired the overall outcome after HSCT, suggesting that *C. difficile* growth was a hallmark of microbiota alteration capable of influencing the success of HSCT.

The main limitation of our study was the use of two different methods of *C. difficile* detection during the 5-year period of our analysis. However, data collection over this long time allowed us to clearly assess the major sensitivity of the two-step algorithm based on GDH followed by PCR for toxin detection.

Our study might prompt future clinical research aimed at minimizing microbial alterations through the use of narrow spectrum antibiotics, reduction of the duration of antibiotic administration and restoring of microbiota diversity with the addition of fiber prebiotics [29] or probiotics [30] to the diet of patients at major risk of CD development.

## Figures and Tables

**Table 1:** Clinical results reported in previously published studies.

Author	Year of Publication	Study Period	Transplant Type	No. of Patients	Patient Features	Incidence of CDI	Diagnostic Methods	GVHD	OS/TRM
Alonso CD [6]	2012	2003–2008	Allo-HSCT, Auto-HSCT	999	Adults	92 (9.2%)	GDH Culture	Related	No difference
Willems L [5]	2012	2004–2007	Allo-HSCT	414	Pediatrics and Adults (4–59)	53 (13%)	EIA toxin A/B GDH Culture	Related	No difference
Trifilio SM [8]	2013	2004–2008	Allo-HSCT, Auto-HSCT	822	Adults	85 (10.3%)	EIA toxin A/B Culture	Related	Increased non-relapse mortality rates in CDI + aGVHD
Bruminhent J [9]	2014	2011–2012	Allo-HSCT, Auto-HSCT	150	Adults	37 (24.7%)	EIA toxin A/B GDH	Not related	No difference
Hosokawa K [10]	2014	2007–2008	Allo-HSCT	167	Adults	17 (10.2%)	EIA toxin A	Not related	No difference
Kamboj M [11]	2014	2005–2010	Allo-HSCT	793	598 Adults, 195 Pediatrics	94 (11.9%)	EIA toxin A/B GDH	Not related	Not indicated
Jain T [12]	2015	2010–2012	Allo-HSCT	150	Adults	25 (16.7%)	Culture PCR	Not related	No difference
Aldrete SdM [13]	2017	2010–2013	Allo-HSCT, Auto-HSCT	650	Adults	86 (13.2%)	PCR	Not related	Higher TRM
Alonso CD [6]	2017	2003–2012	Allo-HSCT (only UCBT)	226	Adults	30 (13.3%)	EIA toxin A/B PCR	Not related	No difference
Scardina T [14]	2017	2009–2014	Allo-HSCT, Auto-HSCT	550	Adults	35 (6%)	EIA toxin A/B PCR	Not Related	No difference
Ford C [15]	2019	2005–2018	Auto-HSCT	472	Adults	33 (7%)	EIA toxin A/B GDH PCR	/	No difference
Amberge S [16]	2020	2004–2015	Allo-HSCT	727	Adults	96 (13%) + 103 (14%) asymptomatic	EIA toxin A/B GDH	Not Related	No difference

aGVHD = acute graft versus host disease; CDI = clostridium difficile infection; EIA = enzyme immunoassays; GDH = *Glutamate dehydrogenase*; HSCT = hematopoietic stem cell transplantation; *PCR* = *polymerase chain reaction*; OS = overall survival; TRM = transplant-related mortality; UCBT = unrelated cord blood transplant.

**Table 2:** Characteristic of patients.

Characteristic	*n* (=481)
Median Age	55 (15–75)
Sex (male/female)	273/208
Diagnosis	
Leukemia and MDS	196 (41%)
Lymphoma	118 (24%)
Multiple myeloma	145 (30%)
Other disease	22 (5%)
Previous infections	177 (37%)
MDR infections	70 (14%)
Gastrointestinal and/or urogenital comorbidities HCT-CI	204 (42%)
0	71 (15%)
1–2	182 (38%)
≥3	228 (47%)
Previous abdominal surgery	172 (36%)
HCV infection	6 (1%)
HBV infection	49 (10%)
Previous Hospitalization	152 (32%)
IgG < 700 mg/dL	207 (43%)
Antibiotic therapy before HSCT	194 (40%)
State disease before HSCT	
Response	287 (60%)
Active	194 (40%)
Type of HSCT	
Autologous HSCT	220 (46%)
Allogeneic HSCT	261 (54%)
HLA identical sibling	48 (19%)
Matched unrelated donor	105 (40%)
Haploidentical donor	47 (18%)
Mismatched unrelated donor	61 (23%)
Stem cell source: PB	462 (96%)
Conditioning regimen	
TBI-based regimen	32 (7%)
Busulfan-based regimen	152 (32%)
RIC	62 (13%)
Other myeloablative regimen	235 (49%)
Antibiotic therapy post-HSCT (n. lines)	
0	107 (22%)
1–2	215 (45%)
≥3	159 (33%)
Mucositis (grade)	
0	45 (9%)
1–2	193 (40%)
3–4	142 (22%)
TPN use	314 (29%)
aGVHD (grade) *	
0	143/261 (55%)
1–2	106/261 (41%)
3–4	12/261 (4%)
CMV reactivation *	147/261 (56%)
Other infections post-HSCT	151 (31%)

aGVHD = acute graft versus host disease; CDI = clostridium difficile infection; CI = confidence interval; HCT-CI = hematopoietic cell transplantation–comorbidity index; HLA = human leukocyte antigen; HSCT = hematopoietic stem cell transplantation; MDS = myelodysplasia; MDR = multidrug-resistant germs; RIC= reduced intensity conditioning; SHR = sub-distribution hazard ratio; TBI = total body irradiation; TPN= total parenteral nutrition. * Calculated on allo-SCT cases.

**Table 3:** Characteristic of patients with CDI.

	2015	2016	2017	2018	2019
Patients with CDI after HSCT (tot = 26)					
Auto-HSCT	0	1	0	5	5
Allo-HSCT	1	1	1	3	9
Toxigenic CD	0	2	1	5	11
CDI presentation (median of days)	6	11	10	10.5	8
Grade of diarrhea					
<4	1	1	-	2	4
4–6	-	-	-	6	7
>6	-	-	1	-	1
Fever > 37.5 °C	1	1	1	1	4
ANC < 1 × 10^9^/L at the time of CDI diagnosis)	1	2	0	3	8
≥grade II acute GVHD (grade 2)	0	1	1	1	3
Gastrointestinal and urogenital comorbidities	1	1	2	7	12
Abdominal surgery	0	0	1	5	10
CD negativization (median of days)	NR	68	10	20.2	11.7
Laboratory test used for CD diagnosis	EIA for toxin	EIA for toxin	EIA for toxin	GDH; PCR for toxin	GDH; PCR for toxin

ANC = absolute neutrophilic count; CD = clostridium difficile; CDI = clostridium difficile infection; EIA = enzyme immunoassays; GDH = *glutamate dehydrogenase*; HSCT = hematopoietic stem cell transplantation; *NR* = *not reached*; *PCR* = *polymerase chain reaction*.

**Table 4:** Univariate analysis of risk factors for incidence of CDI.

Risk Factor	SHR	95% CI	*p*
Sex			
Male	1		
Female	1.13	0.526–2.453	0.74
Age (yr)	1.01	0.983–1.057	0.30
≤60	1		
>60	2.39	1.089–5.257	0.03
Diagnosis			
Leukemia and MDS	1		
Lymphomas	0.44	0.147–1.351	0.15
Myeloma	0.53	0.205–1.368	0.19
Other diagnosis	0.60	0.818–4.518	0.62
Gastrointestinal and urogenital comorbidities	1.89	0.874–4.108	0.01
Previous infections	2.36	1.085–5.137	0.03
Previous MDR infection	1.04	0.362–2.997	0.93
HCT-CI			
HCT-CI 0	1		
HCT-CI 1–2	1.19	0.244–5.841	0.82
HCT-CI ≥ 3	2.87	0.677–12.213	0.15
Previous abdominal surgery	2.96	1.35–6.511	0.007
HCV infection	6.63	1.912–22.842	0.003
HBV infection	1.57	0.552–4.493	0.396
Hospitalization before HSCT	2.55	1.185–5.526	0.017
IgG value pre-HSCT	1.141	0.528–2.466	0.73
Antibiotic therapy before HSCT	8.38	3.868–18.179	0.000
Disease status			
Responsive	1		
Active	0.53	0.224–1.265	0.15
Type of HSCT			
HLA identical donor	1		
Matched unrelated donor	0.95	0.292–3.090	0.93
Haploidentical donor	0.76	0.177–3.338	0.72
Mismatched unrelated donor	0.19	0.022–1.765	1.76
Autologous HSCT	0.55	0.178–1.749	0.31
Conditioning regimen			
TBI-based	1		
Busulfan-based	1.66	0.210–13.104	0.63
Reduced intensity	2.11	0.237–18.822	0.50
Other myeloablative regimen	1.76	0.233–13.351	0.58
Antibiotic therapy post-HSCT (n. lines)			
No lines	1		
1–2 lines	0.66	0.232–1.914	0.45
≥3 lines	1.40	0.531–3.733	0.49
Mucositis (grades)			
0–1–2	1		
3–4	0.68	0.278–1.695	0.41
TPN use	0.51	0.238−1.108	0.09
aGVHD (grades)			
0–1–2	1		
3–4	1.52	0.545–4.28	0.41
CMV reactivation	0.36	0.132–1.006	0.051
Other infections post-HSCT	0.39	0.137–1.134	0.08

aGVHD = acute graft versus host disease; CDI = clostridium difficile infection; CI = confidence interval; HCT-CI = hematopoietic cell transplantation–comorbidity Index; HLA = human leukocyte antigen; HSCT = hematopoietic stem cell transplantation; MDS = myelodysplasia; MDR = multidrug-resistant germs; SHR = sub-distribution hazard ratio; TBI = total body irradiation; TPN= total parenteral nutrition.

**Table 5:** Multivariate analysis of risk factors for CDI.

Factor	SHR	95% CI	*p*
Previous abdominal surgery	3.69	1.614–8.458	0.002
Antibiotic therapy before HSCT	15.09	6.491–35.078	0.000
Previous HCV infection	6.53	1.295–33.016	0.023
Other infections post-HSCT	2.15	0.044–0.529	0.003

CI = confidence interval; HSCT = hematopoietic stem cell transplantation; SHR = sub-distribution hazard ratio.

**Table 6:** Multivariate analysis of risk factors for TRM and OS.

	TRM			OS		
Factor	SHR	*p*	95% CI	SHR	*p*	95% CI
Gastrointestinal comorbidity	1.98	0.008	1.193–3.299	1.50	0.017	1.076–2.096
Previous infections				1.533	0.024	1.058–2.222
Previous Hospitalization				1.84	0.003	1.230–2.757
Disease status				2.23	0.000	1.581–3.170
aGVHD (grades)						
0–3	1					
4	4.07	0.001	1.764–9.392			
Other infections post-HSCT				1.42	0.043	1.011–1.998

aGVHD = acute graft versus host disease; HSCT = hematopoietic stem cell transplantation; OS = overall survival; TRM = transplant-related mortality.
